# Crystal structure of bis­(η^5^-cyclo­penta­dien­yl)(1,4-di-*tert*-butyl­buta-1-en-3-yn-1-yl)zirconium(IV) μ_2_-hydroxido-bis­[tris(penta­fluoro­phen­yl)borate]

**DOI:** 10.1107/S2056989015003710

**Published:** 2015-02-28

**Authors:** Vladimir V. Burlakov, Anke Spannenberg, Perdita Arndt, Uwe Rosenthal

**Affiliations:** a A. N. Nesmeyanov Institute of Organoelement Compounds, Russian Academy of Sciences, Vavilov St. 28, 119991 Moscow, Russian Federation; b Leibniz-Institut für Katalyse e. V. an der Universität Rostock, Albert-Einstein-Strasse 29a, 18059 Rostock, Germany

**Keywords:** crystal structure, zirconocene, buta-1-en-3-yne, borate anion, intra­molecular O—H⋯F hydrogen bonds

## Abstract

Alkyl zirconocene cations have been of considerable inter­est as reactive species in many polymerization processes. In the crystal structure of the title compound, [Zr(C_12_H_19_)(C_5_H_5_)_2_](C_36_HB_2_F_30_O), the [Zr(C_5_H_5_)_2_((*t*-Bu)C=C(H)—C_2_(*t*-Bu))]^+^ cation displays a buta-1-en-3-yne ligand side-on coordinated to a typical bent zirconocene [centroid(cp)—Zr—centroid(cp) = 131.4 (3)°, Zr—C(buta-1-en-3-yne) = 2.255 (3), 2.597 (3) and 2.452 (2) Å]. In the [HO(B(C_6_F_5_)_3_)_2_]^−^ anion, intra­molecular O—H⋯F hydrogen bonds are observed. One *tert*-butyl group in the complex cation is disordered over two sets of sites with occupancies 0.701(4):0.299(4).

## Related literature   

For examples of the coordination of a buta-en-yne ligand to a group IV transition metal atom, see: Erker *et al.* (2004 ▸); Ahlers, Temme, Erker, Fröhlich & Fox (1997 ▸). For complexation of a buta-1-en-3-yne as a bridging ligand between two metallocenes, see: Ahlers, Temme, Erker, Fröhlich & Zippel (1997 ▸); Burlakov *et al.* (2010 ▸). For an example of the structure of the hydroxyl borate anion and its formation, see: Liptau *et al.* (2004 ▸). Stoichimetric reactions of alkyl­zirconocene complexes with B(C_6_F_5_)_3_ have been investigated and different reaction modes (*e.g.* C—C bond coupling or cleavage) and compounds exhibiting inter­esting structural features have been obtained, see: Ahlers, Temme, Erker, Fröhlich & Zippel (1997 ▸); Burlakov *et al.* (2004 ▸).
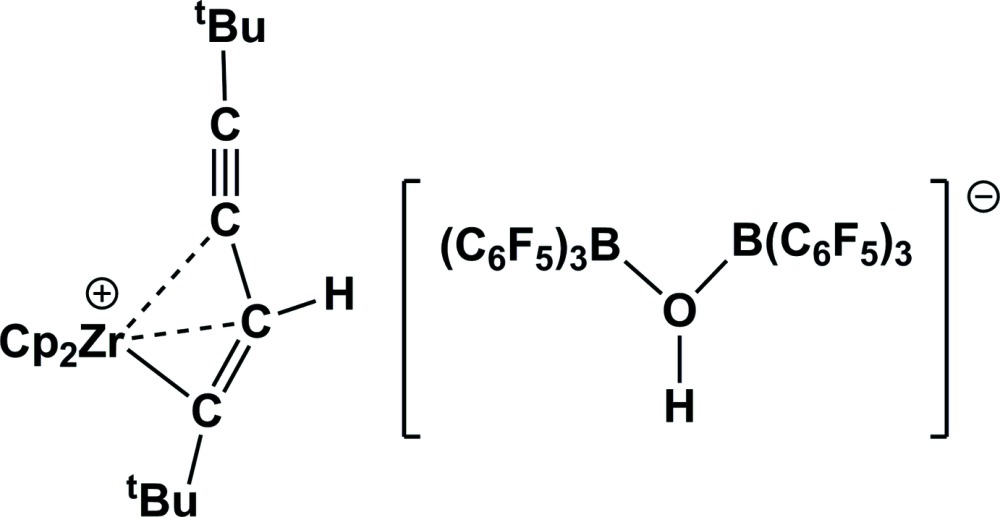


## Experimental   

### Crystal data  

[Zr(C_12_H_19_)(C_5_H_5_)_2_](C_36_HB_2_F_30_O)
*M* *_r_* = 1425.66
Triclinic, 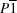

*a* = 12.8896 (5) Å
*b* = 13.6334 (5) Å
*c* = 16.5466 (6) Å
α = 86.730 (3)°
β = 75.389 (3)°
γ = 77.581 (3)°
*V* = 2747.84 (18) Å^3^
*Z* = 2
Mo *K*α radiation
μ = 0.35 mm^−1^
*T* = 200 K
0.25 × 0.25 × 0.17 mm

### Data collection  

Stoe IPDS II diffractometer
Absorption correction: numerical (*X-SHAPE* and *X-RED32*; Stoe & Cie, 2005 ▸) *T*_min_ = 0.810, *T*_max_ = 0.966
47172 measured reflections
13119 independent reflections
8407 reflections with *I* > 2σ(*I*)
*R*_int_ = 0.040

### Refinement  

*R*[*F* ^2^ > 2σ(*F* ^2^)] = 0.037
*wR*(*F* ^2^) = 0.083
*S* = 0.84
13119 reflections
820 parameters
27 restraints
H atoms treated by a mixture of independent and constrained refinement
Δρ_max_ = 0.62 e Å^−3^
Δρ_min_ = −0.41 e Å^−3^

### 

Data collection: *X-AREA* (Stoe & Cie, 2005 ▸); cell refinement: *X-AREA*; data reduction: *X-AREA*; program(s) used to solve structure: *SHELXS97* (Sheldrick, 2008 ▸); program(s) used to refine structure: *SHELXL2014* (Sheldrick, 2015 ▸); molecular graphics: *XP* in *SHELXTL* (Sheldrick, 2008 ▸); software used to prepare material for publication: *SHELXL2014*.

## Supplementary Material

Crystal structure: contains datablock(s) I, New_Global_Publ_Block. DOI: 10.1107/S2056989015003710/nr2057sup1.cif

**Fig. 1.**
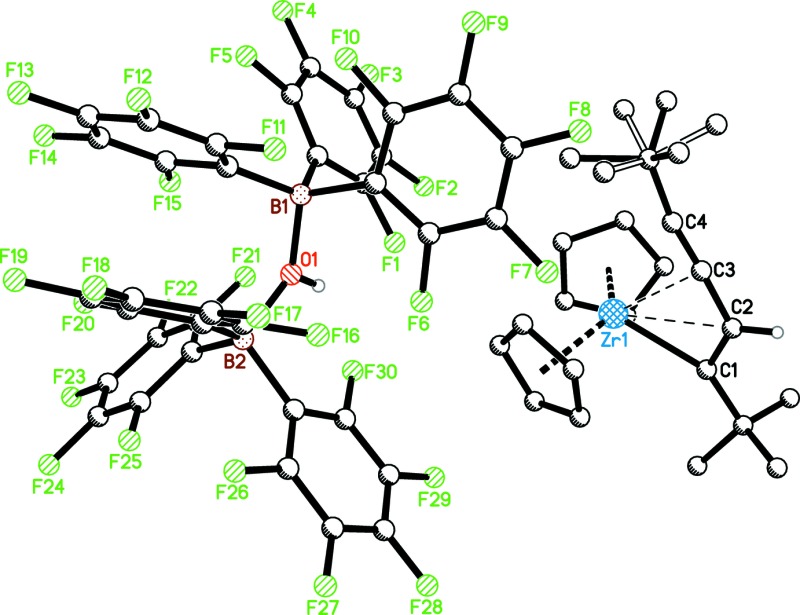
: Ball and stick representation of the molecular structure of the title compound. Zr, B, F, O atoms and C1—C4 are labelled. The minor occupied part of the disordered t-butyl group is shown with open lines. Hydrogen atoms of the cyclopentadienyl ligands and the t-butyl groups are omitted for clarity.

**Fig. 2.**
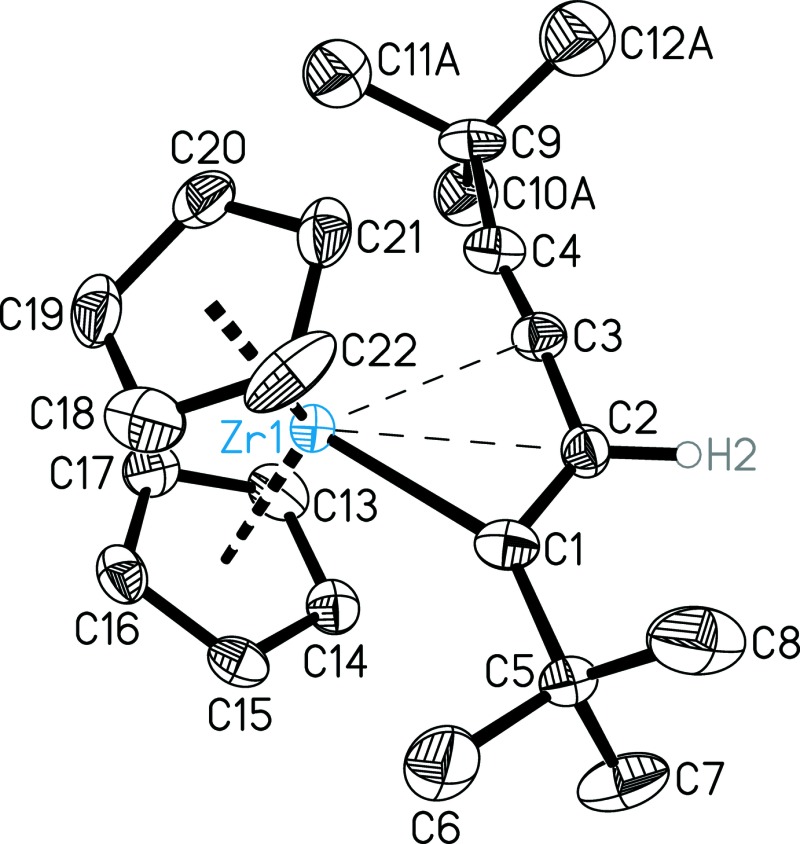
: Molecular structure of the cation with labelling and displacement ellipsoids drawn at 30% probability level. Hydrogen atoms (except H2) and the minor occupied atoms of the disordered t-butyl group are omitted for clarity.

Structure factors: contains datablock(s) I. DOI: 10.1107/S2056989015003710/nr2057Isup2.hkl

t t . DOI: 10.1107/S2056989015003710/nr2057fig1.tif
Ball and stick representation of the mol­ecular structure of the title compound. Zr, B, F, O atoms and C1—C4 are labelled. The minor occupied part of the disordered *t*-butyl group is shown with open lines. Hydrogen atoms of the cyclo­penta­dienyl ligands and the *t*-butyl groups are omitted for clarity.

t . DOI: 10.1107/S2056989015003710/nr2057fig2.tif
Mol­ecular structure of the cation with labelling and displacement ellipsoids drawn at 30% probability level. Hydrogen atoms (except H2) and the minor occupied atoms of the disordered *t*-butyl group are omitted for clarity.

**Fig. 3.**
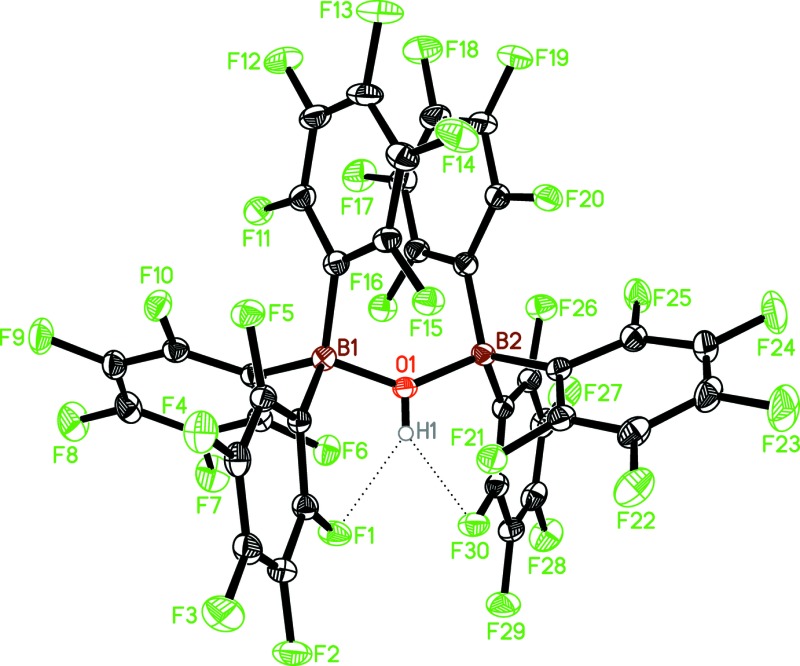
: Molecular structure of the anion with labelling of all non-carbon atoms and displacement ellipsoids drawn at 30% probability level. Hydrogen bonds are shown with dotted lines.

. DOI: 10.1107/S2056989015003710/nr2057fig3.tif
Mol­ecular structure of the anion with labelling of all non-carbon atoms and displacement ellipsoids drawn at 30% probability level. Hydrogen bonds are shown with dotted lines.

CCDC reference: 1050794

Additional supporting information:  crystallographic information; 3D view; checkCIF report

## Figures and Tables

**Table 1:** Hydrogen-bond geometry (, )

*D*H*A*	*D*H	H*A*	*D* *A*	*D*H*A*
O1H1F1	0.76(3)	2.10(3)	2.722(2)	139(2)
O1H1F30	0.76(3)	2.08(3)	2.723(2)	142(2)

## supplementary crystallographic information

### S1. Synthesis and crystallization

B(C_6_F_5_)_3_ (0.376 g, 0.73 mmol) was dissolved in 20 ml of warm (60°C) *n*-hexane under Ar, and the obtained solution was added to Cp_2_Zr[(*t*-Bu)C_4_(*t*-Bu] (0.282 g, 0.73 mmol) in 10 ml of *n*-hexane. At once an orange precipitate was formed, which was separated from the mother liquor by filtration, washed with *n*-hexane, and dried in vacuum. Benzene (5 ml) was added and after one day at RT yellow crystals of the title complex had formed. Yield : 0.187 g (18%); m.p. 150-152°C (dec.) under Ar. Anal. Calcd for C_58_H_30_B_2_F_30_OZr: C, 48.86; H, 2.21%. Found: C, 47.66; H, 2.23%. ^1^H NMR (300 MHz, C_6_D_6_, 297 K): δ = 0.84 (s, 9H, *t*-Bu); 1.25 (s, 9H, *t*-Bu); 5.63 (s, 5H, Cp); 5.63 (s, 5H, Cp) ppm; the signals of C=CH and OH were not detected. ^13^C NMR (75 MHz, C_6_D_6_, 297 K): δ = 29.0, 31.3 (*t*-Bu); 113.7, 114.8 (Cp) ppm; other signals have not been found because of poor solubility. Crystals suitable for X–ray crystal structure analysis were obtained from a saturated *n*-hexane solution at RT after one day.

### S2. Refinement

H1 and H2 could be found from the difference Fourier map and were refined freely. All other H atoms were placed in idealized positions with d(C—H) = 0.95 Å (CH), 0.98 Å (CH_3_) and refined using a riding model with *U*_iso_(H) fixed at 1.2 *U*_eq_(C) for CH and 1.5 *U*_eq_(C) for CH_3_. One *t*-butyl group is disordered over two sites with occupancies 0.701 (4):0.299 (4). SADI and DANG instructions were used to improve the geometry of the disordered *t*-butyl group. Additionally, the isotropic displacement parameters of C10A, C11A, C12A, C10B, C11B and C12B were restrained to be equal (SIMU). SADI restraint was also used for the ordered *t*-butyl group and AFIX 56 for the Cp rings.

### Crystal data

**Table:**

[Zr(C_12_H_19_)(C_5_H_5_)_2_](C_36_HB_2_F_30_O)	*Z* = 2
*M**_r_* = 1425.66	*F*(000) = 1412
Triclinic, *P*1	*D*_x_ = 1.723 Mg m^−^^3^
*a* = 12.8896 (5) Å	Mo *K*α radiation, λ = 0.71073 Å
*b* = 13.6334 (5) Å	Cell parameters from 6318 reflections
*c* = 16.5466 (6) Å	θ = 1.6–28.4°
α = 86.730 (3)°	µ = 0.35 mm^−^^1^
β = 75.389 (3)°	*T* = 200 K
γ = 77.581 (3)°	Prism, yellow
*V* = 2747.84 (18) Å^3^	0.25 × 0.25 × 0.17 mm

### Data collection

**Table:**

Stoe IPDS II diffractometer	13119 independent reflections
Radiation source: fine-focus sealed tube	8407 reflections with *I* > 2σ(*I*)
Graphite monochromator	*R*_int_ = 0.040
ω scans	θ_max_ = 28.0°, θ_min_ = 1.5°
Absorption correction: numerical (*X-SHAPE* and *X-RED32*; Stoe & Cie, 2005)	*h* = −16→16
*T*_min_ = 0.810, *T*_max_ = 0.966	*k* = −17→17
47172 measured reflections	*l* = −21→19

### Refinement

**Table:**

Refinement on *F*^2^	27 restraints
Least-squares matrix: full	Hydrogen site location: mixed
*R*[*F*^2^ > 2σ(*F*^2^)] = 0.037	H atoms treated by a mixture of independent and constrained refinement
*wR*(*F*^2^) = 0.083	*w* = 1/[σ^2^(*F*_o_^2^) + (0.0453*P*)^2^] where *P* = (*F*_o_^2^ + 2*F*_c_^2^)/3
*S* = 0.84	(Δ/σ)_max_ = 0.001
13119 reflections	Δρ_max_ = 0.62 e Å^−^^3^
820 parameters	Δρ_min_ = −0.40 e Å^−^^3^

### Special details

**Table:**

Geometry. All e.s.d.'s (except the e.s.d. in the dihedral angle between two l.s. planes) are estimated using the full covariance matrix. The cell e.s.d.'s are taken into account individually in the estimation of e.s.d.'s in distances, angles and torsion angles; correlations between e.s.d.'s in cell parameters are only used when they are defined by crystal symmetry. An approximate (isotropic) treatment of cell e.s.d.'s is used for estimating e.s.d.'s involving l.s. planes.

### Fractional atomic coordinates and isotropic or equivalent isotropic displacement parameters (Å^2^)

**Table:**

	*x*	*y*	*z*	*U*_iso_*/*U*_eq_	Occ. (<1)
B1	0.62689 (19)	0.20536 (17)	0.29996 (15)	0.0268 (5)	
B2	0.79348 (19)	0.29432 (18)	0.18106 (15)	0.0272 (5)	
C1	0.9852 (2)	0.27187 (19)	0.74447 (15)	0.0425 (6)	
C2	0.9592 (2)	0.18318 (19)	0.75685 (17)	0.0409 (6)	
C3	0.8706 (2)	0.15060 (19)	0.73591 (16)	0.0422 (6)	
C4	0.7936 (2)	0.1297 (2)	0.71818 (18)	0.0510 (7)	
C5	1.0792 (2)	0.30077 (18)	0.77204 (15)	0.0408 (6)	
C6	1.0966 (3)	0.4026 (3)	0.7357 (3)	0.0824 (12)	
H6A	1.1572	0.4206	0.7534	0.124*	
H6B	1.0296	0.4534	0.7557	0.124*	
H6C	1.1143	0.3996	0.6746	0.124*	
C7	1.0480 (3)	0.3083 (3)	0.86594 (19)	0.0805 (11)	
H7A	1.0316	0.2444	0.8902	0.121*	
H7B	0.9831	0.3621	0.8835	0.121*	
H7C	1.1089	0.3232	0.8851	0.121*	
C8	1.1847 (3)	0.2256 (3)	0.7420 (3)	0.1003 (15)	
H8A	1.2432	0.2455	0.7610	0.150*	
H8B	1.2039	0.2232	0.6808	0.150*	
H8C	1.1755	0.1591	0.7646	0.150*	
C9	0.7083 (2)	0.0823 (2)	0.70203 (19)	0.0591 (8)	
C10A	0.6103 (4)	0.0946 (4)	0.7771 (3)	0.0799 (14)*	0.701 (4)
H10A	0.5542	0.0628	0.7656	0.120*	0.701 (4)
H10B	0.6335	0.0628	0.8260	0.120*	0.701 (4)
H10C	0.5799	0.1663	0.7881	0.120*	0.701 (4)
C11A	0.6625 (5)	0.1351 (4)	0.6285 (3)	0.0829 (15)*	0.701 (4)
H11A	0.6061	0.1021	0.6191	0.124*	0.701 (4)
H11B	0.6305	0.2059	0.6423	0.124*	0.701 (4)
H11C	0.7222	0.1305	0.5777	0.124*	0.701 (4)
C12A	0.7591 (5)	−0.0252 (4)	0.6773 (4)	0.1011 (17)*	0.701 (4)
H12A	0.7035	−0.0580	0.6663	0.152*	0.701 (4)
H12B	0.8188	−0.0271	0.6267	0.152*	0.701 (4)
H12C	0.7883	−0.0605	0.7227	0.152*	0.701 (4)
C10B	0.5924 (7)	0.1386 (9)	0.7394 (8)	0.083 (2)*	0.299 (4)
H10D	0.5401	0.1031	0.7263	0.124*	0.299 (4)
H10E	0.5803	0.1426	0.8001	0.124*	0.299 (4)
H10F	0.5819	0.2066	0.7159	0.124*	0.299 (4)
C11B	0.7348 (12)	0.0506 (11)	0.6129 (5)	0.098 (3)*	0.299 (4)
H11D	0.6769	0.0194	0.6040	0.147*	0.299 (4)
H11E	0.7402	0.1095	0.5765	0.147*	0.299 (4)
H11F	0.8049	0.0021	0.5998	0.147*	0.299 (4)
C12B	0.7181 (11)	−0.0169 (7)	0.7545 (8)	0.089 (2)*	0.299 (4)
H12D	0.6637	−0.0539	0.7475	0.134*	0.299 (4)
H12E	0.7918	−0.0584	0.7353	0.134*	0.299 (4)
H12F	0.7049	−0.0005	0.8136	0.134*	0.299 (4)
C13	0.66787 (14)	0.36548 (12)	0.79695 (12)	0.0517 (7)	
H13	0.6272	0.3145	0.8151	0.062*	
C14	0.74940 (15)	0.38772 (13)	0.83266 (10)	0.0475 (7)	
H14	0.7728	0.3542	0.8789	0.057*	
C15	0.78976 (13)	0.46886 (13)	0.78714 (12)	0.0479 (7)	
H15	0.8449	0.4991	0.7976	0.058*	
C16	0.73318 (15)	0.49676 (12)	0.72329 (11)	0.0496 (7)	
H16	0.7439	0.5490	0.6835	0.060*	
C17	0.65784 (14)	0.43287 (15)	0.72935 (11)	0.0517 (7)	
H17	0.6093	0.4349	0.6943	0.062*	
C18	0.9270 (2)	0.41749 (15)	0.56372 (13)	0.0699 (10)	
H18	0.9389	0.4819	0.5730	0.084*	
C19	0.83144 (17)	0.39720 (14)	0.54610 (13)	0.0617 (8)	
H19	0.7681	0.4457	0.5415	0.074*	
C20	0.84714 (16)	0.29177 (15)	0.53661 (13)	0.0552 (7)	
H20	0.7962	0.2573	0.5245	0.066*	
C21	0.95243 (17)	0.24689 (14)	0.54838 (12)	0.0585 (8)	
H21	0.9842	0.1771	0.5455	0.070*	
C22	1.00180 (14)	0.3246 (2)	0.56513 (12)	0.0743 (11)	
H22	1.0724	0.3159	0.5755	0.089*	
C23	0.53417 (16)	0.27195 (15)	0.37623 (13)	0.0259 (4)	
C24	0.56643 (17)	0.31399 (16)	0.43748 (13)	0.0293 (5)	
C25	0.49734 (19)	0.36692 (17)	0.50575 (14)	0.0351 (5)	
C26	0.38647 (19)	0.37978 (17)	0.51604 (14)	0.0369 (5)	
C27	0.34887 (17)	0.33839 (16)	0.45860 (14)	0.0328 (5)	
C28	0.42095 (17)	0.28547 (15)	0.39120 (13)	0.0283 (5)	
C29	0.67605 (17)	0.10434 (15)	0.34827 (13)	0.0290 (5)	
C30	0.77542 (18)	0.08198 (16)	0.36996 (14)	0.0322 (5)	
C31	0.8078 (2)	−0.00110 (18)	0.41655 (16)	0.0416 (6)	
C32	0.7388 (2)	−0.06633 (17)	0.44457 (16)	0.0440 (6)	
C33	0.6386 (2)	−0.04764 (17)	0.42578 (15)	0.0394 (6)	
C34	0.60956 (18)	0.03622 (16)	0.37984 (14)	0.0314 (5)	
C35	0.57823 (16)	0.18643 (16)	0.22220 (13)	0.0286 (5)	
C36	0.59296 (18)	0.09457 (16)	0.18300 (14)	0.0333 (5)	
C37	0.5508 (2)	0.08297 (19)	0.11677 (15)	0.0409 (6)	
C38	0.4893 (2)	0.1647 (2)	0.08566 (16)	0.0435 (6)	
C39	0.47201 (19)	0.25690 (19)	0.12106 (15)	0.0388 (5)	
C40	0.51676 (17)	0.26529 (16)	0.18678 (14)	0.0306 (5)	
C41	0.79519 (17)	0.21253 (16)	0.11253 (13)	0.0292 (5)	
C42	0.84955 (18)	0.11357 (16)	0.11741 (14)	0.0324 (5)	
C43	0.8606 (2)	0.04069 (17)	0.06001 (16)	0.0400 (6)	
C44	0.8142 (2)	0.06373 (19)	−0.00607 (16)	0.0455 (6)	
C45	0.7565 (2)	0.15938 (19)	−0.01305 (14)	0.0400 (6)	
C46	0.74802 (18)	0.23080 (16)	0.04529 (14)	0.0327 (5)	
C47	0.74840 (17)	0.41262 (16)	0.15988 (13)	0.0291 (5)	
C48	0.67159 (18)	0.48323 (16)	0.21216 (14)	0.0325 (5)	
C49	0.64114 (19)	0.58268 (17)	0.19104 (17)	0.0398 (6)	
C50	0.6879 (2)	0.61662 (17)	0.11353 (19)	0.0467 (6)	
C51	0.7663 (2)	0.55051 (19)	0.05963 (16)	0.0452 (6)	
C52	0.79564 (19)	0.45291 (17)	0.08375 (15)	0.0366 (5)	
C53	0.91714 (17)	0.28909 (15)	0.19566 (13)	0.0281 (4)	
C54	1.01596 (18)	0.25210 (16)	0.13889 (14)	0.0335 (5)	
C55	1.11764 (18)	0.24930 (17)	0.15358 (17)	0.0395 (6)	
C56	1.12492 (19)	0.28574 (18)	0.22648 (17)	0.0405 (6)	
C57	1.0302 (2)	0.32661 (18)	0.28417 (16)	0.0385 (5)	
C58	0.93044 (17)	0.32780 (16)	0.26670 (14)	0.0312 (5)	
F1	0.67535 (10)	0.30374 (11)	0.43197 (8)	0.0391 (3)	
F2	0.53833 (13)	0.40589 (12)	0.56093 (9)	0.0520 (4)	
F3	0.31647 (13)	0.43285 (12)	0.58047 (9)	0.0563 (4)	
F4	0.23980 (10)	0.35074 (10)	0.46769 (9)	0.0447 (4)	
F5	0.37404 (10)	0.24591 (10)	0.34052 (8)	0.0384 (3)	
F6	0.84787 (10)	0.14335 (10)	0.34862 (9)	0.0401 (3)	
F7	0.90530 (13)	−0.01638 (12)	0.43567 (11)	0.0603 (4)	
F8	0.76925 (15)	−0.14715 (11)	0.48968 (11)	0.0651 (5)	
F9	0.57076 (13)	−0.11164 (11)	0.45179 (10)	0.0538 (4)	
F10	0.50912 (11)	0.05135 (10)	0.36506 (9)	0.0403 (3)	
F11	0.65509 (11)	0.01120 (9)	0.20768 (9)	0.0411 (3)	
F12	0.57139 (15)	−0.00692 (12)	0.08071 (10)	0.0599 (4)	
F13	0.44963 (15)	0.15406 (14)	0.02003 (10)	0.0652 (5)	
F14	0.41377 (13)	0.33750 (12)	0.09076 (10)	0.0556 (4)	
F15	0.49879 (11)	0.35877 (9)	0.21779 (8)	0.0370 (3)	
F16	0.89746 (11)	0.08421 (9)	0.18083 (9)	0.0406 (3)	
F17	0.91750 (13)	−0.05302 (10)	0.06875 (10)	0.0570 (4)	
F18	0.82371 (17)	−0.00562 (13)	−0.06316 (11)	0.0703 (5)	
F19	0.70551 (15)	0.18250 (12)	−0.07546 (9)	0.0591 (4)	
F20	0.68685 (11)	0.32214 (10)	0.03376 (8)	0.0402 (3)	
F21	0.62084 (11)	0.45871 (9)	0.29049 (8)	0.0379 (3)	
F22	0.56761 (12)	0.64748 (10)	0.24648 (11)	0.0551 (4)	
F23	0.65937 (16)	0.71307 (11)	0.09263 (12)	0.0713 (5)	
F24	0.81554 (16)	0.58198 (12)	−0.01608 (11)	0.0698 (5)	
F25	0.87523 (12)	0.39252 (10)	0.02851 (9)	0.0485 (4)	
F26	1.01811 (11)	0.21525 (11)	0.06457 (8)	0.0436 (3)	
F27	1.20962 (11)	0.21015 (11)	0.09655 (11)	0.0557 (4)	
F28	1.22255 (12)	0.28281 (12)	0.24214 (11)	0.0591 (4)	
F29	1.03531 (13)	0.36563 (13)	0.35548 (10)	0.0558 (4)	
F30	0.84025 (11)	0.37121 (10)	0.32578 (8)	0.0401 (3)	
O1	0.71908 (12)	0.26558 (11)	0.26572 (10)	0.0268 (3)	
Zr1	0.84257 (2)	0.32484 (2)	0.68602 (2)	0.03081 (6)	
H1	0.733 (2)	0.2889 (19)	0.3016 (16)	0.034 (8)*	
H2	1.002 (3)	0.128 (2)	0.782 (2)	0.076 (10)*	

### Atomic displacement parameters (Å^2^)

**Table:**

	*U*^11^	*U*^22^	*U*^33^	*U*^12^	*U*^13^	*U*^23^
B1	0.0244 (11)	0.0262 (11)	0.0289 (12)	−0.0044 (9)	−0.0053 (9)	−0.0019 (10)
B2	0.0282 (12)	0.0297 (12)	0.0230 (11)	−0.0063 (9)	−0.0046 (9)	−0.0017 (9)
C1	0.0434 (14)	0.0499 (14)	0.0320 (13)	−0.0175 (11)	0.0031 (11)	−0.0078 (11)
C2	0.0413 (13)	0.0364 (13)	0.0460 (15)	−0.0085 (11)	−0.0133 (11)	0.0039 (11)
C3	0.0459 (14)	0.0430 (14)	0.0399 (14)	−0.0163 (11)	−0.0089 (11)	−0.0001 (11)
C4	0.0512 (16)	0.0533 (16)	0.0495 (16)	−0.0141 (13)	−0.0085 (13)	−0.0127 (13)
C5	0.0407 (13)	0.0459 (14)	0.0378 (13)	−0.0111 (11)	−0.0106 (11)	−0.0049 (11)
C6	0.076 (2)	0.082 (2)	0.116 (3)	−0.050 (2)	−0.051 (2)	0.029 (2)
C7	0.092 (3)	0.120 (3)	0.0457 (18)	−0.055 (2)	−0.0184 (18)	−0.0064 (19)
C8	0.0468 (19)	0.108 (3)	0.148 (4)	−0.0011 (19)	−0.025 (2)	−0.065 (3)
C9	0.0671 (19)	0.0606 (18)	0.0601 (19)	−0.0289 (15)	−0.0185 (15)	−0.0140 (15)
C13	0.0380 (14)	0.0508 (16)	0.0553 (17)	−0.0099 (12)	0.0110 (12)	−0.0091 (13)
C14	0.0481 (15)	0.0481 (15)	0.0373 (14)	−0.0008 (12)	−0.0005 (12)	−0.0056 (12)
C15	0.0457 (14)	0.0425 (14)	0.0514 (16)	−0.0101 (11)	−0.0007 (13)	−0.0124 (12)
C16	0.0521 (15)	0.0378 (14)	0.0507 (16)	0.0008 (12)	−0.0061 (13)	−0.0016 (12)
C17	0.0350 (13)	0.0566 (16)	0.0587 (18)	0.0051 (12)	−0.0119 (12)	−0.0148 (14)
C18	0.107 (3)	0.068 (2)	0.0350 (15)	−0.046 (2)	0.0052 (17)	0.0036 (14)
C19	0.074 (2)	0.0589 (18)	0.0399 (15)	0.0079 (15)	−0.0128 (15)	0.0104 (13)
C20	0.0660 (19)	0.0672 (19)	0.0375 (14)	−0.0172 (15)	−0.0198 (13)	0.0026 (13)
C21	0.0674 (19)	0.0608 (18)	0.0311 (14)	0.0161 (15)	−0.0073 (13)	−0.0004 (13)
C22	0.0392 (15)	0.161 (4)	0.0248 (13)	−0.034 (2)	−0.0023 (12)	0.0045 (18)
C23	0.0281 (10)	0.0251 (10)	0.0259 (10)	−0.0073 (8)	−0.0078 (8)	0.0020 (8)
C24	0.0273 (10)	0.0323 (11)	0.0275 (11)	−0.0065 (8)	−0.0047 (9)	−0.0009 (9)
C25	0.0433 (13)	0.0378 (12)	0.0262 (11)	−0.0128 (10)	−0.0075 (10)	−0.0047 (9)
C26	0.0396 (13)	0.0321 (11)	0.0304 (12)	−0.0040 (10)	0.0051 (10)	−0.0048 (9)
C27	0.0247 (10)	0.0315 (11)	0.0371 (12)	−0.0041 (9)	−0.0007 (9)	0.0048 (9)
C28	0.0285 (10)	0.0284 (11)	0.0295 (11)	−0.0069 (8)	−0.0092 (9)	0.0016 (9)
C29	0.0314 (11)	0.0277 (11)	0.0259 (11)	−0.0039 (8)	−0.0044 (9)	−0.0038 (9)
C30	0.0324 (11)	0.0310 (11)	0.0317 (11)	−0.0044 (9)	−0.0068 (9)	0.0001 (9)
C31	0.0402 (13)	0.0392 (13)	0.0438 (14)	0.0023 (10)	−0.0164 (11)	0.0015 (11)
C32	0.0561 (16)	0.0296 (12)	0.0404 (14)	0.0006 (11)	−0.0114 (12)	0.0091 (10)
C33	0.0508 (15)	0.0296 (11)	0.0340 (12)	−0.0104 (10)	−0.0021 (11)	0.0012 (10)
C34	0.0316 (11)	0.0322 (11)	0.0296 (11)	−0.0057 (9)	−0.0060 (9)	−0.0036 (9)
C35	0.0259 (10)	0.0310 (11)	0.0286 (11)	−0.0072 (8)	−0.0043 (9)	−0.0033 (9)
C36	0.0358 (12)	0.0303 (11)	0.0338 (12)	−0.0085 (9)	−0.0066 (10)	−0.0031 (9)
C37	0.0457 (14)	0.0435 (14)	0.0365 (13)	−0.0166 (11)	−0.0066 (11)	−0.0139 (11)
C38	0.0430 (14)	0.0616 (17)	0.0336 (13)	−0.0150 (12)	−0.0178 (11)	−0.0094 (12)
C39	0.0348 (12)	0.0491 (14)	0.0335 (12)	−0.0054 (10)	−0.0133 (10)	0.0003 (11)
C40	0.0291 (11)	0.0318 (11)	0.0308 (11)	−0.0061 (9)	−0.0062 (9)	−0.0052 (9)
C41	0.0288 (10)	0.0303 (11)	0.0270 (11)	−0.0066 (8)	−0.0027 (9)	−0.0035 (9)
C42	0.0332 (11)	0.0341 (12)	0.0288 (11)	−0.0080 (9)	−0.0040 (9)	−0.0040 (9)
C43	0.0400 (13)	0.0323 (12)	0.0415 (14)	−0.0039 (10)	0.0005 (11)	−0.0099 (10)
C44	0.0559 (16)	0.0429 (14)	0.0360 (14)	−0.0139 (12)	−0.0011 (12)	−0.0174 (11)
C45	0.0505 (14)	0.0475 (14)	0.0263 (12)	−0.0170 (11)	−0.0105 (11)	−0.0048 (10)
C46	0.0370 (12)	0.0319 (11)	0.0283 (11)	−0.0087 (9)	−0.0048 (9)	−0.0011 (9)
C47	0.0309 (11)	0.0293 (11)	0.0299 (11)	−0.0089 (9)	−0.0102 (9)	−0.0015 (9)
C48	0.0334 (11)	0.0311 (11)	0.0357 (12)	−0.0081 (9)	−0.0121 (10)	−0.0021 (9)
C49	0.0354 (12)	0.0297 (12)	0.0558 (16)	−0.0027 (9)	−0.0159 (11)	−0.0053 (11)
C50	0.0530 (15)	0.0264 (12)	0.0660 (18)	−0.0074 (11)	−0.0272 (14)	0.0097 (12)
C51	0.0579 (16)	0.0403 (13)	0.0418 (14)	−0.0178 (12)	−0.0168 (12)	0.0135 (11)
C52	0.0402 (13)	0.0342 (12)	0.0351 (12)	−0.0090 (10)	−0.0071 (10)	−0.0007 (10)
C53	0.0307 (10)	0.0251 (10)	0.0289 (11)	−0.0082 (8)	−0.0064 (9)	0.0007 (8)
C54	0.0347 (12)	0.0308 (11)	0.0345 (12)	−0.0091 (9)	−0.0052 (10)	−0.0017 (9)
C55	0.0275 (11)	0.0348 (12)	0.0523 (15)	−0.0063 (9)	−0.0029 (10)	−0.0013 (11)
C56	0.0302 (12)	0.0384 (13)	0.0593 (17)	−0.0121 (10)	−0.0196 (11)	0.0052 (12)
C57	0.0418 (13)	0.0424 (13)	0.0398 (13)	−0.0182 (11)	−0.0180 (11)	0.0023 (11)
C58	0.0305 (11)	0.0322 (11)	0.0317 (12)	−0.0112 (9)	−0.0053 (9)	−0.0005 (9)
F1	0.0294 (7)	0.0595 (8)	0.0317 (7)	−0.0124 (6)	−0.0090 (5)	−0.0080 (6)
F2	0.0571 (9)	0.0655 (10)	0.0360 (8)	−0.0182 (8)	−0.0071 (7)	−0.0207 (7)
F3	0.0502 (9)	0.0613 (10)	0.0441 (8)	−0.0036 (7)	0.0105 (7)	−0.0217 (7)
F4	0.0251 (6)	0.0481 (8)	0.0534 (9)	−0.0038 (6)	0.0001 (6)	0.0027 (7)
F5	0.0288 (6)	0.0502 (8)	0.0395 (7)	−0.0119 (6)	−0.0101 (6)	−0.0055 (6)
F6	0.0315 (7)	0.0410 (7)	0.0508 (8)	−0.0096 (6)	−0.0156 (6)	0.0071 (6)
F7	0.0478 (9)	0.0584 (10)	0.0765 (12)	0.0018 (7)	−0.0328 (8)	0.0160 (8)
F8	0.0813 (12)	0.0402 (8)	0.0711 (11)	−0.0026 (8)	−0.0275 (9)	0.0239 (8)
F9	0.0671 (10)	0.0394 (8)	0.0545 (9)	−0.0230 (7)	−0.0066 (8)	0.0117 (7)
F10	0.0374 (7)	0.0407 (7)	0.0463 (8)	−0.0156 (6)	−0.0110 (6)	0.0044 (6)
F11	0.0515 (8)	0.0279 (7)	0.0414 (8)	−0.0040 (6)	−0.0095 (6)	−0.0060 (6)
F12	0.0816 (12)	0.0508 (9)	0.0539 (10)	−0.0201 (8)	−0.0184 (9)	−0.0239 (8)
F13	0.0729 (11)	0.0857 (12)	0.0507 (10)	−0.0167 (9)	−0.0363 (9)	−0.0176 (9)
F14	0.0563 (9)	0.0646 (10)	0.0487 (9)	0.0023 (8)	−0.0310 (8)	0.0012 (8)
F15	0.0427 (7)	0.0303 (7)	0.0386 (7)	−0.0013 (6)	−0.0155 (6)	−0.0034 (6)
F16	0.0434 (8)	0.0339 (7)	0.0436 (8)	−0.0021 (6)	−0.0137 (6)	−0.0019 (6)
F17	0.0633 (10)	0.0335 (8)	0.0652 (11)	0.0039 (7)	−0.0079 (8)	−0.0169 (7)
F18	0.1024 (14)	0.0572 (10)	0.0512 (10)	−0.0145 (10)	−0.0137 (10)	−0.0310 (8)
F19	0.0895 (12)	0.0646 (10)	0.0352 (8)	−0.0253 (9)	−0.0287 (8)	−0.0044 (7)
F20	0.0495 (8)	0.0391 (7)	0.0352 (7)	−0.0069 (6)	−0.0183 (6)	0.0004 (6)
F21	0.0392 (7)	0.0352 (7)	0.0349 (7)	−0.0058 (6)	−0.0008 (6)	−0.0078 (6)
F22	0.0471 (8)	0.0335 (7)	0.0785 (11)	0.0037 (6)	−0.0113 (8)	−0.0148 (7)
F23	0.0904 (13)	0.0339 (8)	0.0910 (14)	−0.0052 (8)	−0.0359 (11)	0.0204 (8)
F24	0.0966 (14)	0.0562 (10)	0.0526 (10)	−0.0241 (9)	−0.0099 (9)	0.0259 (8)
F25	0.0550 (9)	0.0454 (8)	0.0354 (8)	−0.0113 (7)	0.0071 (7)	0.0018 (6)
F26	0.0355 (7)	0.0539 (8)	0.0371 (8)	−0.0088 (6)	0.0011 (6)	−0.0134 (6)
F27	0.0276 (7)	0.0553 (9)	0.0757 (11)	−0.0061 (6)	0.0032 (7)	−0.0135 (8)
F28	0.0360 (8)	0.0666 (10)	0.0839 (12)	−0.0153 (7)	−0.0278 (8)	0.0012 (9)
F29	0.0571 (9)	0.0772 (11)	0.0469 (9)	−0.0301 (8)	−0.0231 (8)	−0.0057 (8)
F30	0.0365 (7)	0.0507 (8)	0.0343 (7)	−0.0152 (6)	−0.0026 (6)	−0.0134 (6)
O1	0.0281 (8)	0.0298 (8)	0.0243 (8)	−0.0091 (6)	−0.0065 (6)	−0.0033 (6)
Zr1	0.02742 (11)	0.03248 (11)	0.03073 (12)	−0.00538 (8)	−0.00494 (8)	0.00140 (9)

### Geometric parameters (Å, º)

**Table:**

B1—O1	1.560 (3)	C20—C21	1.4200
B1—C35	1.622 (3)	C20—Zr1	2.522 (2)
B1—C29	1.641 (3)	C20—H20	0.9500
B1—C23	1.658 (3)	C21—C22	1.4200
B2—O1	1.567 (3)	C21—Zr1	2.5147 (19)
B2—C41	1.628 (3)	C21—H21	0.9500
B2—C47	1.642 (3)	C22—Zr1	2.4779 (18)
B2—C53	1.658 (3)	C22—H22	0.9500
C1—C2	1.314 (3)	C23—C24	1.382 (3)
C1—C5	1.529 (4)	C23—C28	1.390 (3)
C1—Zr1	2.255 (3)	C24—F1	1.361 (2)
C2—C3	1.434 (4)	C24—C25	1.378 (3)
C2—Zr1	2.597 (3)	C25—F2	1.347 (3)
C2—H2	0.96 (3)	C25—C26	1.369 (3)
C3—C4	1.197 (4)	C26—F3	1.340 (2)
C3—Zr1	2.452 (2)	C26—C27	1.367 (4)
C4—C9	1.473 (4)	C27—F4	1.350 (2)
C4—Zr1	2.855 (3)	C27—C28	1.381 (3)
C5—C8	1.504 (4)	C28—F5	1.344 (3)
C5—C7	1.507 (4)	C29—C30	1.383 (3)
C5—C6	1.520 (4)	C29—C34	1.390 (3)
C6—H6A	0.9800	C30—F6	1.354 (3)
C6—H6B	0.9800	C30—C31	1.382 (3)
C6—H6C	0.9800	C31—F7	1.342 (3)
C7—H7A	0.9800	C31—C32	1.372 (4)
C7—H7B	0.9800	C32—F8	1.339 (3)
C7—H7C	0.9800	C32—C33	1.371 (4)
C8—H8A	0.9800	C33—F9	1.344 (3)
C8—H8B	0.9800	C33—C34	1.373 (3)
C8—H8C	0.9800	C34—F10	1.348 (3)
C9—C11B	1.494 (7)	C35—C40	1.387 (3)
C9—C12A	1.505 (5)	C35—C36	1.398 (3)
C9—C10B	1.516 (7)	C36—F11	1.349 (3)
C9—C10A	1.519 (5)	C36—C37	1.370 (3)
C9—C11A	1.558 (5)	C37—F12	1.339 (3)
C9—C12B	1.564 (7)	C37—C38	1.377 (4)
C10A—H10A	0.9800	C38—F13	1.339 (3)
C10A—H10B	0.9800	C38—C39	1.367 (4)
C10A—H10C	0.9800	C39—F14	1.340 (3)
C11A—H11A	0.9800	C39—C40	1.374 (3)
C11A—H11B	0.9800	C40—F15	1.352 (2)
C11A—H11C	0.9800	C41—C46	1.384 (3)
C12A—H12A	0.9800	C41—C42	1.388 (3)
C12A—H12B	0.9800	C42—F16	1.347 (3)
C12A—H12C	0.9800	C42—C43	1.374 (3)
C10B—H10D	0.9800	C43—F17	1.349 (3)
C10B—H10E	0.9800	C43—C44	1.364 (4)
C10B—H10F	0.9800	C44—F18	1.340 (3)
C11B—H11D	0.9800	C44—C45	1.370 (4)
C11B—H11E	0.9800	C45—F19	1.347 (3)
C11B—H11F	0.9800	C45—C46	1.378 (3)
C12B—H12D	0.9800	C46—F20	1.355 (3)
C12B—H12E	0.9800	C47—C48	1.381 (3)
C12B—H12F	0.9800	C47—C52	1.392 (3)
C13—C17	1.4200	C48—F21	1.355 (3)
C13—C14	1.4200	C48—C49	1.379 (3)
C13—Zr1	2.4982 (17)	C49—F22	1.347 (3)
C13—H13	0.9500	C49—C50	1.373 (4)
C14—C15	1.4200	C50—F23	1.337 (3)
C14—Zr1	2.5277 (17)	C50—C51	1.369 (4)
C14—H14	0.9500	C51—F24	1.346 (3)
C15—C16	1.4200	C51—C52	1.368 (3)
C15—Zr1	2.5186 (18)	C52—F25	1.347 (3)
C15—H15	0.9500	C53—C58	1.379 (3)
C16—C17	1.4200	C53—C54	1.391 (3)
C16—Zr1	2.4832 (18)	C54—F26	1.346 (3)
C16—H16	0.9500	C54—C55	1.384 (3)
C17—Zr1	2.4705 (18)	C55—F27	1.342 (3)
C17—H17	0.9500	C55—C56	1.362 (4)
C18—C22	1.4200	C56—F28	1.339 (3)
C18—C19	1.4200	C56—C57	1.375 (4)
C18—Zr1	2.4624 (19)	C57—F29	1.343 (3)
C18—H18	0.9500	C57—C58	1.384 (3)
C19—C20	1.4200	C58—F30	1.362 (2)
C19—Zr1	2.4900 (19)	O1—H1	0.76 (3)
C19—H19	0.9500		
			
O1—B1—C35	107.31 (17)	F3—C26—C27	120.8 (2)
O1—B1—C29	109.15 (17)	F3—C26—C25	120.7 (2)
C35—B1—C29	115.91 (18)	C27—C26—C25	118.5 (2)
O1—B1—C23	107.78 (16)	F4—C27—C26	119.25 (19)
C35—B1—C23	113.25 (17)	F4—C27—C28	119.9 (2)
C29—B1—C23	103.14 (17)	C26—C27—C28	120.8 (2)
O1—B2—C41	106.53 (16)	F5—C28—C27	115.20 (19)
O1—B2—C47	108.88 (16)	F5—C28—C23	121.45 (18)
C41—B2—C47	116.31 (19)	C27—C28—C23	123.3 (2)
O1—B2—C53	107.65 (17)	C30—C29—C34	113.41 (19)
C41—B2—C53	111.91 (17)	C30—C29—B1	127.16 (19)
C47—B2—C53	105.27 (17)	C34—C29—B1	119.08 (19)
C2—C1—C5	124.4 (3)	F6—C30—C31	114.6 (2)
C2—C1—Zr1	89.37 (18)	F6—C30—C29	121.46 (19)
C5—C1—Zr1	146.15 (18)	C31—C30—C29	123.9 (2)
C1—C2—C3	128.2 (3)	F7—C31—C32	120.4 (2)
C1—C2—Zr1	60.23 (16)	F7—C31—C30	120.0 (2)
C3—C2—Zr1	68.00 (15)	C32—C31—C30	119.6 (2)
C1—C2—H2	122 (2)	F8—C32—C33	120.6 (2)
C3—C2—H2	109 (2)	F8—C32—C31	120.2 (2)
Zr1—C2—H2	177 (2)	C33—C32—C31	119.2 (2)
C4—C3—C2	175.8 (3)	F9—C33—C32	119.9 (2)
C4—C3—Zr1	96.9 (2)	F9—C33—C34	120.9 (2)
C2—C3—Zr1	79.16 (15)	C32—C33—C34	119.2 (2)
C3—C4—C9	167.8 (3)	F10—C34—C33	116.2 (2)
C3—C4—Zr1	58.49 (17)	F10—C34—C29	119.13 (19)
C9—C4—Zr1	133.6 (2)	C33—C34—C29	124.6 (2)
C8—C5—C7	111.1 (3)	C40—C35—C36	112.9 (2)
C8—C5—C6	107.9 (3)	C40—C35—B1	120.82 (18)
C7—C5—C6	108.6 (3)	C36—C35—B1	126.22 (19)
C8—C5—C1	111.2 (2)	F11—C36—C37	115.9 (2)
C7—C5—C1	108.2 (2)	F11—C36—C35	120.2 (2)
C6—C5—C1	109.9 (2)	C37—C36—C35	123.8 (2)
C5—C6—H6A	109.5	F12—C37—C36	120.4 (2)
C5—C6—H6B	109.5	F12—C37—C38	119.7 (2)
H6A—C6—H6B	109.5	C36—C37—C38	119.9 (2)
C5—C6—H6C	109.5	F13—C38—C39	120.7 (2)
H6A—C6—H6C	109.5	F13—C38—C37	120.0 (2)
H6B—C6—H6C	109.5	C39—C38—C37	119.3 (2)
C5—C7—H7A	109.5	F14—C39—C38	120.0 (2)
C5—C7—H7B	109.5	F14—C39—C40	121.0 (2)
H7A—C7—H7B	109.5	C38—C39—C40	118.9 (2)
C5—C7—H7C	109.5	F15—C40—C39	116.06 (19)
H7A—C7—H7C	109.5	F15—C40—C35	118.8 (2)
H7B—C7—H7C	109.5	C39—C40—C35	125.2 (2)
C5—C8—H8A	109.5	C46—C41—C42	113.3 (2)
C5—C8—H8B	109.5	C46—C41—B2	126.49 (19)
H8A—C8—H8B	109.5	C42—C41—B2	120.2 (2)
C5—C8—H8C	109.5	F16—C42—C43	115.7 (2)
H8A—C8—H8C	109.5	F16—C42—C41	119.9 (2)
H8B—C8—H8C	109.5	C43—C42—C41	124.3 (2)
C4—C9—C11B	111.2 (6)	F17—C43—C44	120.2 (2)
C4—C9—C12A	108.3 (3)	F17—C43—C42	120.1 (2)
C4—C9—C10B	113.8 (6)	C44—C43—C42	119.7 (2)
C11B—C9—C10B	117.1 (7)	F18—C44—C43	121.1 (2)
C4—C9—C10A	110.3 (3)	F18—C44—C45	120.1 (3)
C12A—C9—C10A	114.1 (4)	C43—C44—C45	118.9 (2)
C4—C9—C11A	111.2 (3)	F19—C45—C44	119.9 (2)
C12A—C9—C11A	107.6 (4)	F19—C45—C46	120.2 (2)
C10A—C9—C11A	105.2 (4)	C44—C45—C46	119.9 (2)
C4—C9—C12B	103.7 (6)	F20—C46—C45	115.1 (2)
C11B—C9—C12B	106.0 (7)	F20—C46—C41	121.0 (2)
C10B—C9—C12B	103.5 (7)	C45—C46—C41	123.9 (2)
C9—C10A—H10A	109.5	C48—C47—C52	112.9 (2)
C9—C10A—H10B	109.5	C48—C47—B2	127.54 (19)
H10A—C10A—H10B	109.5	C52—C47—B2	119.34 (19)
C9—C10A—H10C	109.5	F21—C48—C49	114.5 (2)
H10A—C10A—H10C	109.5	F21—C48—C47	121.25 (19)
H10B—C10A—H10C	109.5	C49—C48—C47	124.2 (2)
C9—C11A—H11A	109.5	F22—C49—C50	119.6 (2)
C9—C11A—H11B	109.5	F22—C49—C48	120.6 (2)
H11A—C11A—H11B	109.5	C50—C49—C48	119.8 (2)
C9—C11A—H11C	109.5	F23—C50—C51	121.1 (2)
H11A—C11A—H11C	109.5	F23—C50—C49	120.3 (2)
H11B—C11A—H11C	109.5	C51—C50—C49	118.6 (2)
C9—C12A—H12A	109.5	F24—C51—C52	120.3 (2)
C9—C12A—H12B	109.5	F24—C51—C50	120.0 (2)
H12A—C12A—H12B	109.5	C52—C51—C50	119.7 (2)
C9—C12A—H12C	109.5	F25—C52—C51	116.6 (2)
H12A—C12A—H12C	109.5	F25—C52—C47	118.73 (19)
H12B—C12A—H12C	109.5	C51—C52—C47	124.7 (2)
C9—C10B—H10D	109.5	C58—C53—C54	113.1 (2)
C9—C10B—H10E	109.5	C58—C53—B2	121.19 (18)
H10D—C10B—H10E	109.5	C54—C53—B2	125.7 (2)
C9—C10B—H10F	109.5	F26—C54—C55	115.2 (2)
H10D—C10B—H10F	109.5	F26—C54—C53	121.0 (2)
H10E—C10B—H10F	109.5	C55—C54—C53	123.8 (2)
C9—C11B—H11D	109.5	F27—C55—C56	119.5 (2)
C9—C11B—H11E	109.5	F27—C55—C54	120.4 (2)
H11D—C11B—H11E	109.5	C56—C55—C54	120.1 (2)
C9—C11B—H11F	109.5	F28—C56—C55	120.9 (2)
H11D—C11B—H11F	109.5	F28—C56—C57	120.0 (2)
H11E—C11B—H11F	109.5	C55—C56—C57	119.1 (2)
C9—C12B—H12D	109.5	F29—C57—C56	120.2 (2)
C9—C12B—H12E	109.5	F29—C57—C58	121.0 (2)
H12D—C12B—H12E	109.5	C56—C57—C58	118.7 (2)
C9—C12B—H12F	109.5	F30—C58—C53	119.30 (19)
H12D—C12B—H12F	109.5	F30—C58—C57	115.6 (2)
H12E—C12B—H12F	109.5	C53—C58—C57	125.1 (2)
C17—C13—C14	108.0	B1—O1—B2	140.58 (18)
C17—C13—Zr1	72.33 (7)	B1—O1—H1	110.4 (19)
C14—C13—Zr1	74.73 (7)	B2—O1—H1	109.0 (19)
C17—C13—H13	126.0	C1—Zr1—C3	63.23 (9)
C14—C13—H13	126.0	C1—Zr1—C18	100.98 (9)
Zr1—C13—H13	118.8	C3—Zr1—C18	136.21 (8)
C15—C14—C13	108.0	C1—Zr1—C17	135.79 (7)
C15—C14—Zr1	73.30 (6)	C3—Zr1—C17	118.55 (8)
C13—C14—Zr1	72.45 (7)	C18—Zr1—C17	101.29 (8)
C15—C14—H14	126.0	C1—Zr1—C22	77.84 (7)
C13—C14—H14	126.0	C3—Zr1—C22	103.64 (9)
Zr1—C14—H14	120.1	C18—Zr1—C22	33.4
C14—C15—C16	108.0	C17—Zr1—C22	134.23 (7)
C14—C15—Zr1	74.01 (6)	C1—Zr1—C16	116.49 (8)
C16—C15—Zr1	72.14 (7)	C3—Zr1—C16	143.21 (7)
C14—C15—H15	126.0	C18—Zr1—C16	80.53 (7)
C16—C15—H15	126.0	C17—Zr1—C16	33.3
Zr1—C15—H15	119.7	C22—Zr1—C16	112.32 (8)
C17—C16—C15	108.0	C1—Zr1—C19	132.07 (8)
C17—C16—Zr1	72.85 (7)	C3—Zr1—C19	131.40 (8)
C15—C16—Zr1	74.88 (7)	C18—Zr1—C19	33.3
C17—C16—H16	126.0	C17—Zr1—C19	82.58 (6)
C15—C16—H16	126.0	C22—Zr1—C19	55.1
Zr1—C16—H16	118.2	C16—Zr1—C19	78.42 (6)
C13—C17—C16	108.0	C1—Zr1—C13	110.14 (7)
C13—C17—Zr1	74.47 (7)	C3—Zr1—C13	89.50 (7)
C16—C17—Zr1	73.84 (7)	C18—Zr1—C13	133.39 (7)
C13—C17—H17	126.0	C17—Zr1—C13	33.2
C16—C17—H17	126.0	C22—Zr1—C13	166.77 (8)
Zr1—C17—H17	117.7	C16—Zr1—C13	54.9
C22—C18—C19	108.0	C19—Zr1—C13	114.67 (6)
C22—C18—Zr1	73.89 (7)	C1—Zr1—C21	91.49 (8)
C19—C18—Zr1	74.41 (8)	C3—Zr1—C21	83.51 (7)
C22—C18—H18	126.0	C18—Zr1—C21	55.0
C19—C18—H18	126.0	C17—Zr1—C21	132.38 (6)
Zr1—C18—H18	117.7	C22—Zr1—C21	33.0
C20—C19—C18	108.0	C16—Zr1—C21	131.87 (5)
C20—C19—Zr1	74.79 (8)	C19—Zr1—C21	54.7
C18—C19—Zr1	72.28 (7)	C13—Zr1—C21	151.26 (7)
C20—C19—H19	126.0	C1—Zr1—C15	85.15 (7)
C18—C19—H19	126.0	C3—Zr1—C15	120.69 (7)
Zr1—C19—H19	118.8	C18—Zr1—C15	96.22 (6)
C19—C20—C21	108.0	C17—Zr1—C15	54.8
C19—C20—Zr1	72.31 (7)	C22—Zr1—C15	117.67 (7)
C21—C20—Zr1	73.34 (7)	C16—Zr1—C15	33.0
C19—C20—H20	126.0	C19—Zr1—C15	107.37 (6)
C21—C20—H20	126.0	C13—Zr1—C15	54.5
Zr1—C20—H20	120.2	C21—Zr1—C15	149.83 (7)
C20—C21—C22	108.0	C1—Zr1—C20	124.24 (8)
C20—C21—Zr1	73.91 (7)	C3—Zr1—C20	98.49 (8)
C22—C21—Zr1	72.06 (7)	C18—Zr1—C20	54.9
C20—C21—H21	126.0	C17—Zr1—C20	99.78 (6)
C22—C21—H21	126.0	C22—Zr1—C20	54.7
Zr1—C21—H21	119.9	C16—Zr1—C20	108.20 (6)
C18—C22—C21	108.0	C19—Zr1—C20	32.9
C18—C22—Zr1	72.70 (7)	C13—Zr1—C20	122.54 (7)
C21—C22—Zr1	74.90 (8)	C21—Zr1—C20	32.8
C18—C22—H22	126.0	C15—Zr1—C20	139.66 (6)
C21—C22—H22	126.0	C1—Zr1—C14	81.68 (7)
Zr1—C22—H22	118.3	C3—Zr1—C14	90.97 (7)
C24—C23—C28	112.72 (19)	C18—Zr1—C14	128.86 (6)
C24—C23—B1	120.32 (18)	C17—Zr1—C14	54.7
C28—C23—B1	126.75 (19)	C22—Zr1—C14	145.77 (7)
F1—C24—C25	115.3 (2)	C16—Zr1—C14	54.6
F1—C24—C23	118.95 (18)	C19—Zr1—C14	132.40 (5)
C25—C24—C23	125.7 (2)	C13—Zr1—C14	32.8
F2—C25—C26	120.7 (2)	C21—Zr1—C14	172.66 (6)
F2—C25—C24	120.5 (2)	C15—Zr1—C14	32.7
C26—C25—C24	118.8 (2)	C20—Zr1—C14	153.94 (6)
			
C5—C1—C2—C3	177.6 (2)	B1—C35—C36—F11	−1.9 (3)
Zr1—C1—C2—C3	0.0 (3)	C40—C35—C36—C37	−0.3 (3)
C5—C1—C2—Zr1	177.6 (3)	B1—C35—C36—C37	−179.3 (2)
C1—C2—C3—Zr1	0.0 (3)	F11—C36—C37—F12	0.3 (3)
Zr1—C3—C4—C9	174.4 (13)	C35—C36—C37—F12	177.8 (2)
C2—C1—C5—C8	51.8 (4)	F11—C36—C37—C38	−178.0 (2)
Zr1—C1—C5—C8	−132.4 (3)	C35—C36—C37—C38	−0.5 (4)
C2—C1—C5—C7	−70.5 (3)	F12—C37—C38—F13	0.5 (4)
Zr1—C1—C5—C7	105.3 (3)	C36—C37—C38—F13	178.8 (2)
C2—C1—C5—C6	171.1 (3)	F12—C37—C38—C39	−177.7 (2)
Zr1—C1—C5—C6	−13.1 (4)	C36—C37—C38—C39	0.6 (4)
C3—C4—C9—C11B	−94.7 (15)	F13—C38—C39—F14	0.8 (4)
Zr1—C4—C9—C11B	78.7 (6)	C37—C38—C39—F14	179.0 (2)
C3—C4—C9—C12A	−31.3 (15)	F13—C38—C39—C40	−178.1 (2)
Zr1—C4—C9—C12A	142.1 (3)	C37—C38—C39—C40	0.1 (4)
C3—C4—C9—C10B	130.5 (14)	F14—C39—C40—F15	−0.3 (3)
Zr1—C4—C9—C10B	−56.1 (7)	C38—C39—C40—F15	178.5 (2)
C3—C4—C9—C10A	94.3 (14)	F14—C39—C40—C35	−179.9 (2)
Zr1—C4—C9—C10A	−92.4 (4)	C38—C39—C40—C35	−1.0 (4)
C3—C4—C9—C11A	−149.4 (13)	C36—C35—C40—F15	−178.45 (18)
Zr1—C4—C9—C11A	24.0 (4)	B1—C35—C40—F15	0.6 (3)
C3—C4—C9—C12B	18.8 (15)	C36—C35—C40—C39	1.1 (3)
Zr1—C4—C9—C12B	−167.8 (5)	B1—C35—C40—C39	−179.9 (2)
C17—C13—C14—C15	0.0	O1—B2—C41—C46	111.3 (2)
Zr1—C13—C14—C15	65.19 (7)	C47—B2—C41—C46	−10.2 (3)
C17—C13—C14—Zr1	−65.19 (7)	C53—B2—C41—C46	−131.3 (2)
C13—C14—C15—C16	0.0	O1—B2—C41—C42	−69.9 (2)
Zr1—C14—C15—C16	64.63 (7)	C47—B2—C41—C42	168.51 (18)
C13—C14—C15—Zr1	−64.63 (7)	C53—B2—C41—C42	47.5 (2)
C14—C15—C16—C17	0.0	C46—C41—C42—F16	−178.54 (18)
Zr1—C15—C16—C17	65.86 (6)	B2—C41—C42—F16	2.5 (3)
C14—C15—C16—Zr1	−65.86 (6)	C46—C41—C42—C43	2.7 (3)
C14—C13—C17—C16	0.0	B2—C41—C42—C43	−176.3 (2)
Zr1—C13—C17—C16	−66.79 (7)	F16—C42—C43—F17	−0.6 (3)
C14—C13—C17—Zr1	66.79 (7)	C41—C42—C43—F17	178.2 (2)
C15—C16—C17—C13	0.0	F16—C42—C43—C44	179.7 (2)
Zr1—C16—C17—C13	67.21 (7)	C41—C42—C43—C44	−1.4 (4)
C15—C16—C17—Zr1	−67.21 (7)	F17—C43—C44—F18	0.3 (4)
C22—C18—C19—C20	0.0	C42—C43—C44—F18	179.9 (2)
Zr1—C18—C19—C20	−66.83 (7)	F17—C43—C44—C45	179.7 (2)
C22—C18—C19—Zr1	66.83 (7)	C42—C43—C44—C45	−0.7 (4)
C18—C19—C20—C21	0.0	F18—C44—C45—F19	2.7 (4)
Zr1—C19—C20—C21	−65.16 (7)	C43—C44—C45—F19	−176.7 (2)
C18—C19—C20—Zr1	65.16 (7)	F18—C44—C45—C46	−179.2 (2)
C19—C20—C21—C22	0.0	C43—C44—C45—C46	1.3 (4)
Zr1—C20—C21—C22	−64.48 (7)	F19—C45—C46—F20	−0.3 (3)
C19—C20—C21—Zr1	64.48 (7)	C44—C45—C46—F20	−178.4 (2)
C19—C18—C22—C21	0.0	F19—C45—C46—C41	178.1 (2)
Zr1—C18—C22—C21	67.17 (8)	C44—C45—C46—C41	0.1 (4)
C19—C18—C22—Zr1	−67.17 (8)	C42—C41—C46—F20	176.43 (18)
C20—C21—C22—C18	0.0	B2—C41—C46—F20	−4.7 (3)
Zr1—C21—C22—C18	−65.70 (7)	C42—C41—C46—C45	−2.0 (3)
C20—C21—C22—Zr1	65.70 (7)	B2—C41—C46—C45	176.9 (2)
O1—B1—C23—C24	−46.7 (2)	O1—B2—C47—C48	11.0 (3)
C35—B1—C23—C24	−165.22 (18)	C41—B2—C47—C48	131.4 (2)
C29—B1—C23—C24	68.7 (2)	C53—B2—C47—C48	−104.1 (2)
O1—B1—C23—C28	139.0 (2)	O1—B2—C47—C52	−174.3 (2)
C35—B1—C23—C28	20.5 (3)	C41—B2—C47—C52	−54.0 (3)
C29—B1—C23—C28	−105.6 (2)	C53—B2—C47—C52	70.5 (3)
C28—C23—C24—F1	178.08 (18)	C52—C47—C48—F21	−176.8 (2)
B1—C23—C24—F1	3.0 (3)	B2—C47—C48—F21	−1.9 (4)
C28—C23—C24—C25	−1.9 (3)	C52—C47—C48—C49	2.2 (3)
B1—C23—C24—C25	−177.0 (2)	B2—C47—C48—C49	177.1 (2)
F1—C24—C25—F2	1.0 (3)	F21—C48—C49—F22	0.8 (3)
C23—C24—C25—F2	−179.0 (2)	C47—C48—C49—F22	−178.2 (2)
F1—C24—C25—C26	−179.50 (19)	F21—C48—C49—C50	179.3 (2)
C23—C24—C25—C26	0.5 (3)	C47—C48—C49—C50	0.3 (4)
F2—C25—C26—F3	1.1 (3)	F22—C49—C50—F23	−1.1 (4)
C24—C25—C26—F3	−178.4 (2)	C48—C49—C50—F23	−179.6 (2)
F2—C25—C26—C27	−179.7 (2)	F22—C49—C50—C51	176.9 (2)
C24—C25—C26—C27	0.8 (3)	C48—C49—C50—C51	−1.6 (4)
F3—C26—C27—F4	−0.6 (3)	F23—C50—C51—F24	−0.5 (4)
C25—C26—C27—F4	−179.7 (2)	C49—C50—C51—F24	−178.5 (2)
F3—C26—C27—C28	178.6 (2)	F23—C50—C51—C52	178.4 (3)
C25—C26—C27—C28	−0.6 (3)	C49—C50—C51—C52	0.3 (4)
F4—C27—C28—F5	−2.7 (3)	F24—C51—C52—F25	0.5 (4)
C26—C27—C28—F5	178.11 (19)	C50—C51—C52—F25	−178.4 (2)
F4—C27—C28—C23	178.12 (18)	F24—C51—C52—C47	−178.7 (2)
C26—C27—C28—C23	−1.1 (3)	C50—C51—C52—C47	2.4 (4)
C24—C23—C28—F5	−176.94 (18)	C48—C47—C52—F25	177.3 (2)
B1—C23—C28—F5	−2.3 (3)	B2—C47—C52—F25	1.9 (3)
C24—C23—C28—C27	2.2 (3)	C48—C47—C52—C51	−3.5 (4)
B1—C23—C28—C27	176.85 (19)	B2—C47—C52—C51	−178.9 (2)
O1—B1—C29—C30	10.9 (3)	O1—B2—C53—C58	−43.2 (2)
C35—B1—C29—C30	132.2 (2)	C41—B2—C53—C58	−159.96 (19)
C23—B1—C29—C30	−103.5 (2)	C47—B2—C53—C58	72.8 (2)
O1—B1—C29—C34	−176.40 (18)	O1—B2—C53—C54	140.4 (2)
C35—B1—C29—C34	−55.1 (3)	C41—B2—C53—C54	23.6 (3)
C23—B1—C29—C34	69.2 (2)	C47—B2—C53—C54	−103.6 (2)
C34—C29—C30—F6	−176.21 (19)	C58—C53—C54—F26	−177.27 (19)
B1—C29—C30—F6	−3.2 (3)	B2—C53—C54—F26	−0.6 (3)
C34—C29—C30—C31	1.6 (3)	C58—C53—C54—C55	3.2 (3)
B1—C29—C30—C31	174.7 (2)	B2—C53—C54—C55	179.9 (2)
F6—C30—C31—F7	−1.2 (3)	F26—C54—C55—F27	−1.2 (3)
C29—C30—C31—F7	−179.2 (2)	C53—C54—C55—F27	178.3 (2)
F6—C30—C31—C32	177.4 (2)	F26—C54—C55—C56	179.3 (2)
C29—C30—C31—C32	−0.6 (4)	C53—C54—C55—C56	−1.2 (4)
F7—C31—C32—F8	−1.5 (4)	F27—C55—C56—F28	−0.2 (3)
C30—C31—C32—F8	179.9 (2)	C54—C55—C56—F28	179.4 (2)
F7—C31—C32—C33	178.5 (2)	F27—C55—C56—C57	179.4 (2)
C30—C31—C32—C33	−0.1 (4)	C54—C55—C56—C57	−1.1 (4)
F8—C32—C33—F9	−0.9 (4)	F28—C56—C57—F29	1.4 (3)
C31—C32—C33—F9	179.1 (2)	C55—C56—C57—F29	−178.1 (2)
F8—C32—C33—C34	179.6 (2)	F28—C56—C57—C58	−179.4 (2)
C31—C32—C33—C34	−0.4 (4)	C55—C56—C57—C58	1.1 (3)
F9—C33—C34—F10	1.9 (3)	C54—C53—C58—F30	176.71 (18)
C32—C33—C34—F10	−178.6 (2)	B2—C53—C58—F30	−0.1 (3)
F9—C33—C34—C29	−177.8 (2)	C54—C53—C58—C57	−3.3 (3)
C32—C33—C34—C29	1.6 (4)	B2—C53—C58—C57	179.9 (2)
C30—C29—C34—F10	178.12 (19)	F29—C57—C58—F30	0.5 (3)
B1—C29—C34—F10	4.5 (3)	C56—C57—C58—F30	−178.7 (2)
C30—C29—C34—C33	−2.2 (3)	F29—C57—C58—C53	−179.5 (2)
B1—C29—C34—C33	−175.8 (2)	C56—C57—C58—C53	1.3 (4)
O1—B1—C35—C40	−69.8 (2)	C35—B1—O1—B2	−16.1 (3)
C29—B1—C35—C40	167.98 (18)	C29—B1—O1—B2	110.2 (3)
C23—B1—C35—C40	49.0 (3)	C23—B1—O1—B2	−138.4 (2)
O1—B1—C35—C36	109.1 (2)	C41—B2—O1—B1	−20.7 (3)
C29—B1—C35—C36	−13.1 (3)	C47—B2—O1—B1	105.5 (3)
C23—B1—C35—C36	−132.1 (2)	C53—B2—O1—B1	−140.9 (2)
C40—C35—C36—F11	177.04 (18)		

### Hydrogen-bond geometry (Å, º)

**Table:**

*D*—H···*A*	*D*—H	H···*A*	*D*···*A*	*D*—H···*A*
O1—H1···F1	0.76 (3)	2.10 (3)	2.722 (2)	139 (2)
O1—H1···F30	0.76 (3)	2.08 (3)	2.723 (2)	142 (2)
