# Supplementary figures and images for: Crystal structure of bis­(η5-cyclo­penta­dien­yl)(1,4-di-tert-butyl­buta-1-en-3-yn-1-yl)zirconium(IV) μ2-hydroxido-bis­[tris(penta­fluoro­phen­yl)borate]
Source: Acta Crystallogr E Crystallogr Commun. 2015 Feb 28;71(Pt 3):m71–2. doi: 10.1107/S2056989015003710 (PMC4350704; doi:10.1107/S2056989015003710)

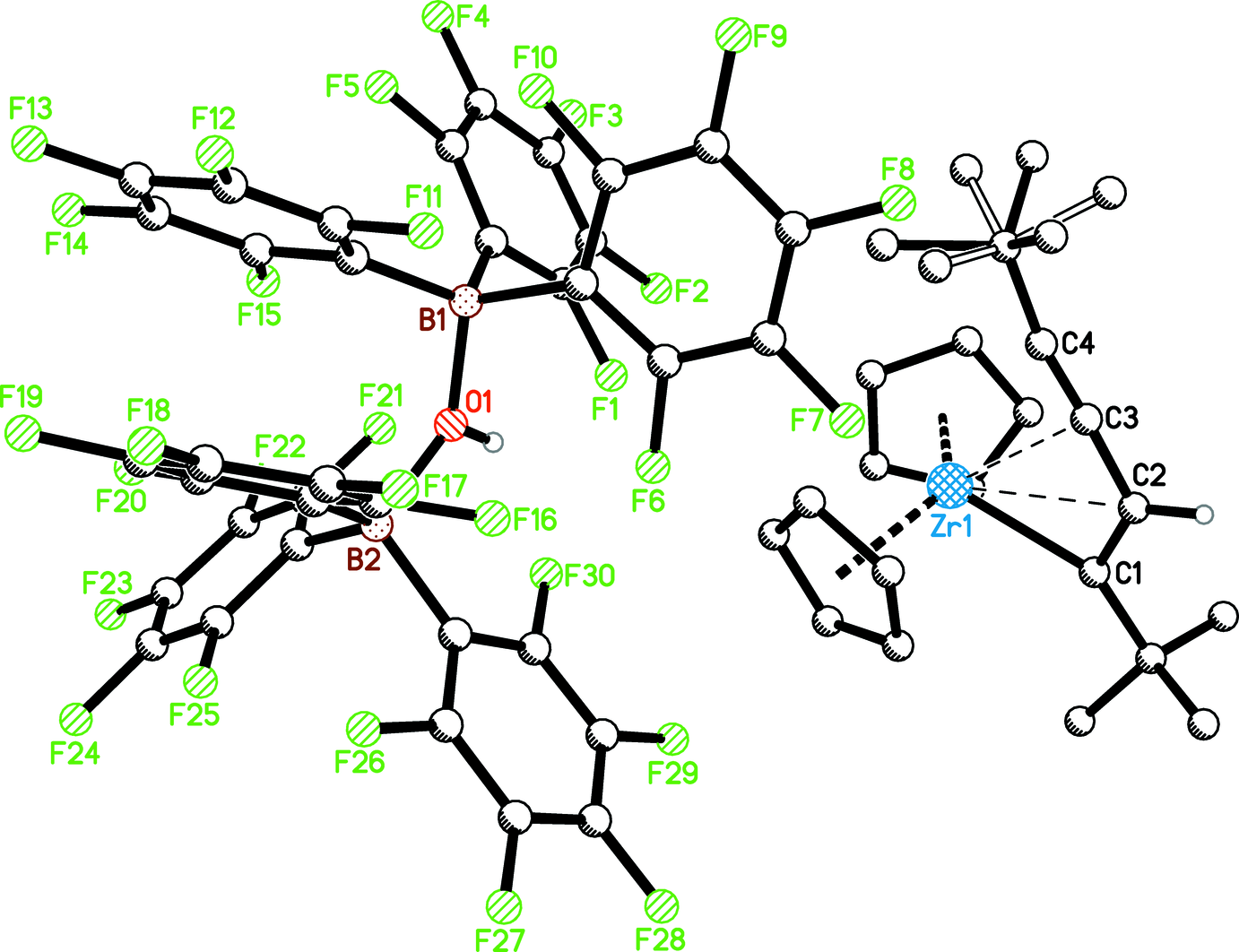

Supplement: Supplementary file 3 [file e-71-00m71-fig1.tif]

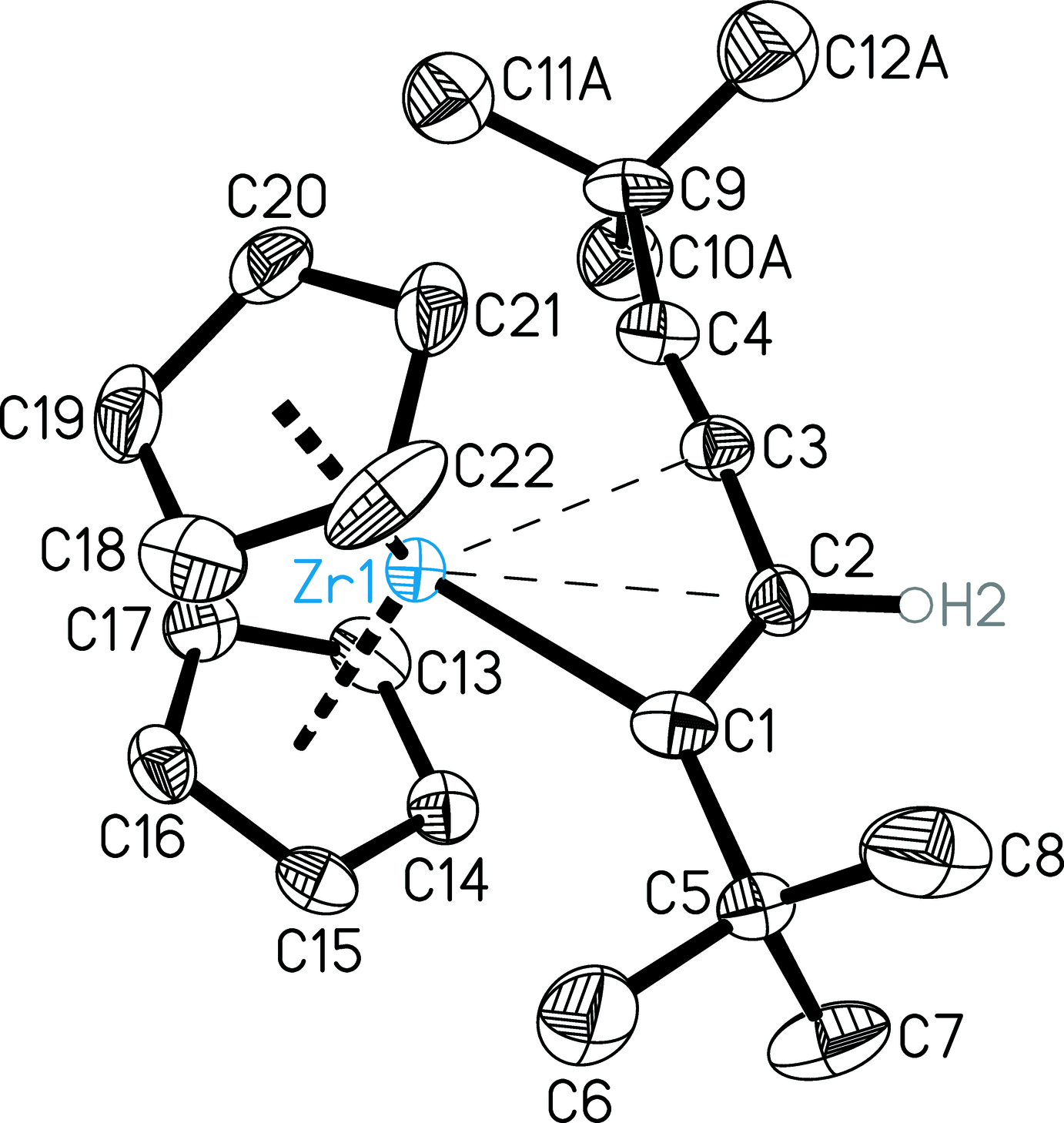

Supplement: Supplementary file 4 [file e-71-00m71-fig2.tif]

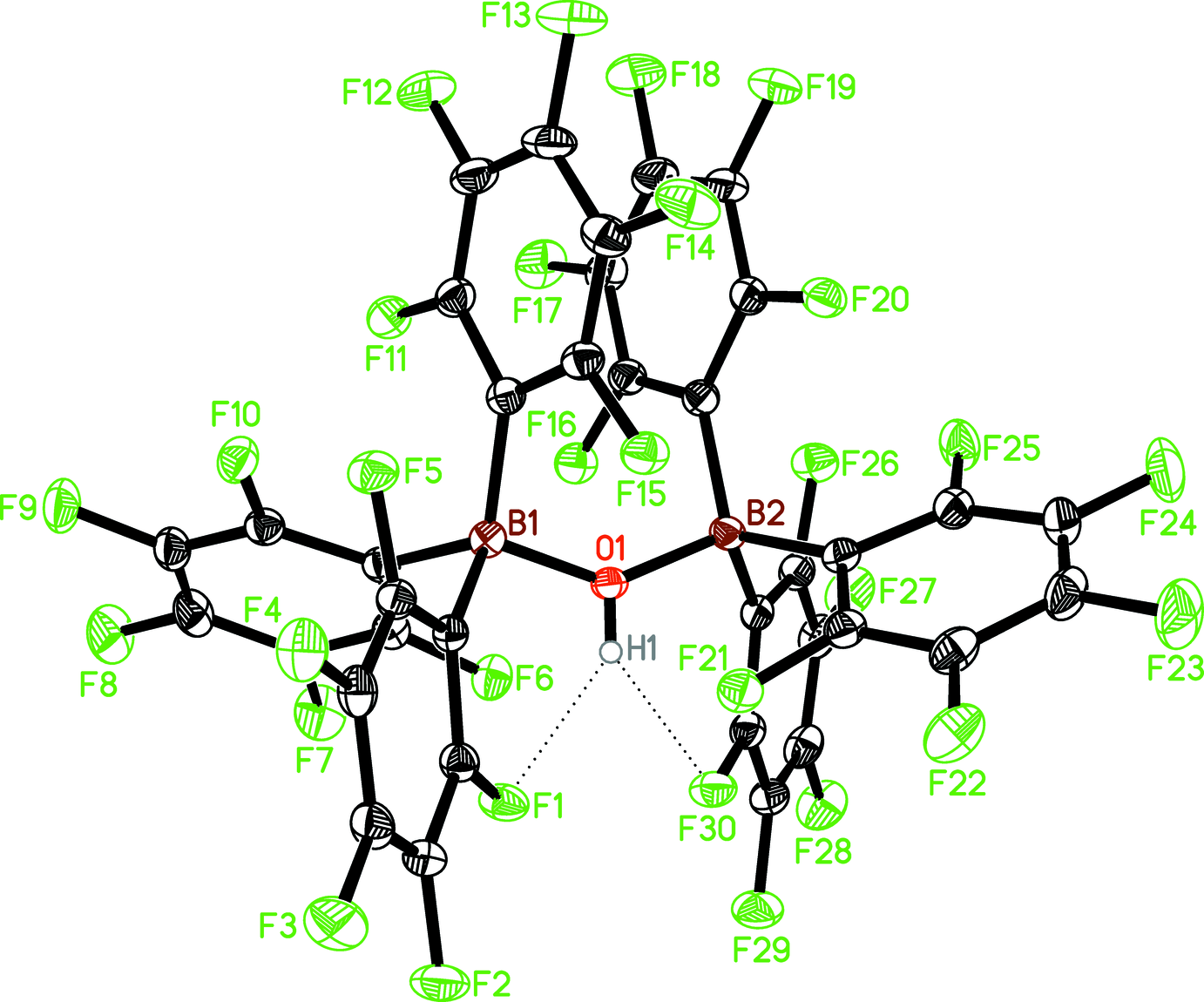

Supplement: Supplementary file 5 [file e-71-00m71-fig3.tif]
